# Characterising rhamnolipid production in *Burkholderia thailandensis* E264, a non-pathogenic producer

**DOI:** 10.1007/s00253-016-7564-y

**Published:** 2016-05-05

**Authors:** Scott J. Funston, Konstantina Tsaousi, Michelle Rudden, Thomas J. Smyth, Paul S. Stevenson, Roger Marchant, Ibrahim M. Banat

**Affiliations:** 1 School of Biomedical Sciences, University of Ulster, Coleraine, Northern Ireland BT52 1SA UK; 2 Department of Life Sciences, Institute of Technology Sligo, County Sligo, Ireland; 3 Research and Development, Unilever, Port Sunlight, Wirral, Liverpool, CH63 3JW UK

**Keywords:** Biosurfactants, Rhamnolipids, *Burkholderia thailandensis*, Gene expression, Di-rhamnolipids, qPCR

## Abstract

*Burkholderia thailandensis* E264 is a rhamnolipid (RL)-producing gram-negative bacterium first isolated from the soils and stagnant waters of central and north-eastern Thailand. Growth of *B. thailandensis* E264 under two different incubation temperatures (25 and 30 °C) resulted in a significantly higher dry cell biomass production at 30 °C (7.71 g/l) than at 25 °C (4.75 g/l) after 264 h; however, incubation at the lower temperature resulted in consistently higher concentration of RL production throughout the growth period. After 264 h, the concentration of crude RL extract for the 25 °C culture was 2.79 g/l compared to 1.99 g/l for the 30 °C culture. Overall RL production concentration after 264 h was 0.258 g/g dry cell biomass (DCB) for the 30 °C culture compared to 0.587 g/g DCB for the 25 °C culture. Real-time PCR (qPCR) was also used to analyse expression of the RL biosynthesis genes throughout the incubation period at 25 °C showing that the expression of the *rhlA*, *rhlB* and *rhlC* genes is continuous. During the log and early stationary phases of growth, expression levels remain low and are increased upon entry to the late stationary phase. *B. thailandensis* E264 produces mostly di-RLs and the Di-RL C14-C14 in most abundance (41.88 %). Fermentations were also carried out in small-scale bioreactors (4 l working volume) under controlled conditions, and results showed that RL production was maintained. Our findings show that *B. thailandensis* E264 has excellent potential for industrial scale RL production.

## Introduction

Rhamnolipids (RLs) were first described in 1949 and were shown to be produced by *Pseudomonas aeruginosa* (Jarvis and Johnson 1949). Since then, there has been a large amount of research carried out into RL production in *P. aeruginosa* and it has been shown that RL production plays an important role in a number of important cell functions. For example, RLs are responsible for maintaining cell-free channels between macrocolonies during the formation of 3D biofilms by *P. aeruginosa*; this is thought to allow the flow of nutrients to the lower layers of the biofilm when cell density increases (Davey et al. 2003). It has also been shown that RL production is involved in swarming motility (Verstraeten et al. 2008).

RLs have gained major interest from industry in recent years due to their effective tensioactive properties, biodegradability, low toxicity and stability throughout a range of temperatures and pH values (Abdel-Mawgoud et al. 2010). These properties make RLs extremely promising compounds for industrial use due to their structural diversity and potential for use in areas such as enhanced oil recovery, environmental bioremediation, food processing and pharmaceuticals (Mukherjee et al. 2006; Banat et al. 2010). As the majority of surfactants currently used today are produced from petrochemical feedstock’s, biosurfactants, and in particular, RLs are seen as a sustainable and environmentally friendly alternative. One of the main problems in using *P. aeruginosa* to produce RLs on an industrial scale is the high cost of production; this is mainly due to low production concentration and the increased safety measures required when working with *P. aeruginosa*, a known opportunistic pathogen. In addition, many consumers tend to avoid using products that have been produced from a pathogenic source. One way in which this problem can be avoided is to use non-pathogenic bacteria for RL production.

Until recently, the production of RLs was thought to be limited to the *Pseudomonas* spp.; however, in recent years, a number of studies have shown that many other gram-negative bacteria are also capable of RL production including a number of species of *Burkholderia* such as *Burkholderia thailandensis*, *Burkholderia glumae*, *Burkholderia kururiensis* and *Burkholderia plantarii* (Dubeau et al. 2009; Hoermann et al. 2010; Costa et al. 2011; Tavares et al. 2013; Wittgens et al. 2011).

*B. thailandensis* E264 is a gram-negative bacterium first isolated from the soils and stagnant waters of central and north-eastern Thailand (Brett et al. 1998). It has a high physiological and genetic similarity to *Burkholderia pseudomallei* which is known to cause melioidosis in both humans and animals; however, *B. thailandensis* E264 was shown to have significantly reduced levels of pathogenicity and is considered an essentially non-pathogenic, biosafety level 1 organism (Brett et al. 1998, Koh et al. 2012). *B. thailandensis* was shown to produce RLs when grown on both glycerol and canola oil with the Di-RL C14-C14 produced in most abundance (Dubeau et al. 2009). This bacterium has good potential as an alternative to *P. aeruginosa* in industrial level RL production; however, very little is known about the RL biosynthesis pathways and regulation systems. As the *B. thailandensis* E264 genome has been fully sequenced, however, it has been highlighted that it contains two identical gene clusters containing copies of the *rhlA*, *rhlB* and *rhlC* genes (Dubeau et al. 2009). This is unlike the corresponding gene orthologs in *P. aeruginosa* where there is a single operon containing the *rhlA* and *rhlB* genes (as well as two regulator genes, *rhlI* and *rhlR*). The *rhlC* gene is located at a different site in the *P. aeruginosa* genome although it is still co-expressed with the other *rhl* genes (Perfumo et al. 2013). As it is not known whether expression of the two *rhl* gene operons in *B. thailandensis* are controlled by the same promoter system, further work is required in order to fully understand how RL synthesis is regulated in *B. thailandensis* and whether it can be exploited for overproduction. This study aimed to characterise RL production in *B. thailandensis* E264 to analyse its potential as a RL-producing organism of industrial interest.

## Methods and materials

### Bacterial strains

*B. thailandensis* E264 was obtained from the American Type Culture Collection (ATCC 700388™) (Brett et al. 1998). Nutrient broth (NB) and nutrient agar were used for the maintenance and storage of this strain. For RL production experiments, NB containing 4 % glycerol (*v*/*v*) was used as the culture medium.

### Bacterial growth conditions

For shake flask RL production experiments, a seed culture of *B. thailandensis* E264 was grown in 100 ml NB+ 4 % glycerol at 30 °C with 200 rpm rotary shaking for 24 h. Ten millilitres of this seed culture was then added to 90 ml sterile NB+ 4 % glycerol to in a 1-l Erlenmeyer flask, and cultures were incubated at either 25 or 30 °C with 200 rpm rotary shaking. Cell growth was monitored by measuring the optical density (OD) of the culture at 600 nm throughout the fermentation. For each measurement, a 100-μl sample of the culture was taken and diluted 1:10 with sterile NB+ 4 % glycerol. All shake flask growth experiments were carried out in biological triplicate to ensure reproducibility.

For analysis under controlled fermentation conditions, *B. thailandensis* E264 was grown in the Biostat B bioreactor (Sartorius) at a working volume of 4 l. An incubation temperature of 25 °C and a dissolved oxygen (DO_2_) level of 20 % were maintained throughout the fermentation. Stirrer speed was programmed to vary between 50 and 500 rpm in order to maintain DO_2_ levels at 20 %. pH was monitored throughout the fermentation, and regular OD measurements were taken to track the biomass levels of *B. thailandensis* E264. Samples were taken at 24-h intervals to monitor RL production. All fermentations using the Biostat B fermenter system were carried out in duplicate to ensure reproducibility.

### Rhamnolipid extraction

In order to gain a complete RL production profile, one 100-ml culture of *B. thailandensis* E264 was grown for each time point in the shake flask experiments (each set of fermentations was seeded from the same seed culture), and 50-ml cell culture samples were taken for each time point in the bioreactor experiments. At each time point, the cell culture was removed and centrifuged at 17,500×*g* for 15 min to remove cell biomass. Extraction methods were based on previous work by Smyth et al. (2010). Supernatant was collected, acidified to pH 2.0 with concentrated HCl and extracted three times with an equal volume of ethyl acetate. The organic phase was collected and dried by adding 0.5 g MgSO_4_ per 100 ml ethyl acetate. The solution was filtered and rotary evaporated to obtain a crude extract containing RLs. All crude extracts were measured gravimetrically before further analysis. Solid phase extraction (SPE) was carried out using Strata SI-1 Silica (55 μm, 70 A) Giga tubes (Phenomenex®) to remove any unwanted impurities from the RL extracts. Purified RL samples were then measured gravimetrically prior to further analysis.

### Glycerol quantification from cell-free culture supernatant

The method used was based on a spectrophotometric method for the determination of free glycerol in biodiesel (Bondioli et al. 2005). A series of glycerol reactions were performed for the formation of formaldehyde by periodate. 3,5-Diacetyl-1,4-dihydrolutidine is produced by the Hantzsch reaction of formaldehyde with acetylacetone. In brief, a sample of the cell-free culture supernatant was diluted in 1:1 *v*/*v* water/ethanol solution. One millilitres of the diluted sample was transferred to a 10-ml test tube, and 1.2 ml of 0.2 M acetylacetone solution and an equal volume of 10 mM sodium periodate solution were added. For the preparation of the acetylacetone and periodate solution, a mixture of 1:1 *v*/*v* of acetic acid (1.6 M) and ammonium acetate (4.0 M) was used. The OD of the resulting product was then measured at 410 nm. A standard curve was used to quantify the glycerol present in each sample.

### Generating a dry cell biomass v optical density standard curve

A standard curve was used in order to readily convert optical density values (OD_600_) to the corresponding dry cell biomass concentration (g/l). A 10 % series dilution was prepared using a *B. thailandensis* E264 cell culture, and OD values were recorded for each 50 ml solution before being centrifuged at 17,500×*g* for 15 min. The resulting cell supernatant was discarded. The pellet formed after centrifugation was used to determine the dry cell weight by weighing the pellet before and after drying at 105 °C until a constant weight was observed (NFTA 2.2.2.5). Dry cell weights were used along with their corresponding OD_600_ values to generate a standard curve.

### Analysing differences in *rhlA*, *rhlB* and *rhlC* amino acid sequences between *B. thailandensis* E264 and *P. aeruginosa* PAO1 using BLASTp alignments

In order to look at the differences between gene orthologs in *P. aeruginosa* and *B. thailandensis*, the amino acid sequences from the *rhlA*, *rhlB* and *rhlC* genes in *B. thailandensis* were aligned with the *P. aeruginosa* genome using the BLASTp algorithm. All DNA and amino acid sequences used for BLAST alignment (NCBI) were obtained from either the *Pseudomonas* genome database or the *Burkholderia* genome database (Winsor et al. 2011; Winsor et al. 2008). The BLASTp algorithm was used to search and align non-redundant protein sequences within the specific genome (i.e. *P. aeruginosa* taxid: 287).

### Separation of rhamnolipid congeners using LC-QToF-MS

Liquid chromatography-hybrid quadrupole time-of-flight mass spectrometry (LC-QToF-MS) of RL crude extract was carried out after SPE purification. For LC separation, the following parameters were used: Static phase, Aglient poroshell SB-C3, 2.1 × 100 mm, particle size 2.7 μm. Mobile phase 1, H_2_O (4 mM ammonium acetate), and mobile phase 2, MeCN, were used for chromatographic separation as follows: 0–17 min 50–70 % mobile phase 2, 17.0–17.5 min 70 % mobile phase 2, 17.5–18.0 min: 70–50 % mobile phase 2, 18–20 min: 50 % mobile phase 2. SPE purification was carried out using Strata SI-1 silica (55 μm, 70 A) 2 g/12 ml Giga Tubes. CHCl_3_ was used to clean the column before the sample was added. The sample was then dissolved in CHCl_3_ and added to the column, and pure CHCl_3_ was run through the column to clean and remove any unwanted products from the sample. Finally, the purified RL was eluted using a 1:1 *v*/*v* solution of CHCl_3_/MeOH.

### Rhamnolipid analysis

Individual RL congeners were identified in the crude extract by electrospray ionisation tandem mass spectrometry (ESI-MS) as described previously (Smyth et al. 2010). This was carried out using an LCQ quadrupole ion trap mass spectrometer (Finnigan MAT, San Jose, CA, USA).

### RNA extraction and cDNA synthesis

At various time points during growth experiments, cell pellets were taken and used for RNA extraction. One millilitres of cell culture was taken and centrifuged at 16,500×*g* for 2 min, the supernatant was then aspirated and the dry cell pellet was immediately moved to −80 °C storage. RNA was isolated from cell pellets using the GeneJET RNA Purification Kit (Thermo Scientific) according to the manufacturer’s instructions. A double DNAse digest was carried out on the purified RNA (Qiagen, RNase free DNase set) once during the extraction process and a second in-solution digest on the final extracted RNA. All extracted RNA was quantified and checked for purity using the NanoDrop 1000 (Thermo Scientific), and samples containing less than 80 ng/μl were discarded and a repeat RNA extraction was carried out. Samples were considered essentially pure with an A260 nm/280 nm ratio of ∼2.0. RNA samples were tested for contaminating DNA using 16S PCR (27F-5′AGAGTTTGATCCTGGCTCAG 3′, 1492R-5′GGTTACCTTGTTACGACTT 3′) with *Taq* DNA polymerase (Invitrogen) and considered DNA free with a clear gel electrophoresis image showing no amplification. All extracted RNA was also checked for structural integrity using gel electrophoresis. RNA was considered intact and viable when the 23S ribosomal RNA (rRNA) and 16S rRNA subunits were observed as clear and distinct bands with no sign of degradation (i.e. smears in the gel).

Complementary DNA (cDNA) was synthesised using Superscript II reverse transcriptase (Invitrogen), random primers (Promega) and 500 ng RNA according to manufacturer’s instructions (final reaction volume 20 μl). Negative control reactions were also carried out using nuclease-free H_2_O (NFW) instead of reverse transcriptase (−RT controls). Primers specific to the *rhlA*, *rhlB* and *rhlC* genes were designed along with reference gene primers for *rpoB*, *rpoD* and *gyrB* using Primer3Plus (Table 1). All primers were optimised and screened for efficiency prior to use in real-time PCR (qPCR) experiments. Primer sets with an efficiency of ∼2.0 we deemed acceptable for use.

**Table 1:** Oligonucleotide primers used for qPCR in this study

Target gene	Forward/reverse	Sequence (5′-3′)	Primer name	Target size (bp)	PCR efficiency
*rhl*A	F	GCGAGTACATTCTGACGAAGG	rhlA1q F	87	1.967
	R	ACACCGACAGCAGGAAACTC	rhlA1q R		
*rhl*B	F	ACGAGGCGATGGCTAAAG	rhlBq F	119	1.936
	R	ATGCGTGCAGAACACCAC	rhlBq R		
*rhl*C	F	ATGCATCACGGGTGGTTG	rhlC1q F	114	1.907
	R	CCATATCGTCAGCAGATTCG	rhlC1q R		
*gyr*B	F	ATCCGACGATCTTCCACATC	gyrBq F	92	1.954
	R	CAGCACGTTTTCGTTGTAGC	gyrBq R		
*rpo*D	F	ACCGTCGTGGCTACAAATTC	rpoDq F	117	1.919
	R	TCGTCTCGATCATGTGAACC	rpoDq R		
*rpo*B	F	TTCGTGAGCTATGCGTTGTC	rpoBq F	175	2.292
	R	TTTCGCCCATGTACACTTCC	rpoBq R		

### Real-time PCR analysis

All qPCR was carried out using the LightCycler 480 (Roche) in a final reaction volume of 10 μl consisting of 5 μl SYBR Green I Mastermix (Roche), 1 μM each primer, 1 μl template cDNA and 2 μl NFW. The following heat cycling parameters were used for each qPCR reaction: 95 °C 5 min × 1 cycle, 95 °C 10 s, 59 °C 10 s, 72 °C 10 s × 50 cycles. After the qPCR reaction, melt curve analysis and gel electrophoresis were carried out to ensure no un-specific amplification had occurred. All reactions were carried out in technical triplicate including negative controls (−RT control and no template controls) and positive controls (using 50 ng gDNA instead of cDNA). qPCR was carried out in biological triplicate (*n* = 3) for each time point to analyse expression levels of the *rhlA*, *rhlB* and *rhlC* genes. Relative gene expression levels were calculated using calibrator-normalised quantification corrected for PCR efficiency. Calibration curves were used to calculate the efficiency of each primer set using the equation, *E* = 10^[−1 / slope]^ (Pfaffl 2001) and were generated using the LightCycler® 480 (software version 1.5). Standards for the cDNA calibration curves were made using fivefold serial dilutions of pooled cDNA ranging through five orders of magnitude. The geometric means of a combination of commonly used reference genes (*rpo*B, *rpo*D and *gyr*B) were used to calculate normalised relative expression levels, and all were relative to the time zero calibrator according to the method previously described by Perfumo et al. (2013).

## Results

### Increased crude extract concentration under lower incubation temperature

*B. thailandensis* E264 was grown for a period of 264 h under two different incubation temperatures, one at 25 °C and one at 30 °C both with 200 rpm rotary shaking. Throughout this period, the cell biomass was monitored (OD_600_) and used as an indicator of cell growth and was converted to dry cell biomass (g/l) using a standard curve. A 50-ml sample of each culture was harvested after every 24 h throughout the study and extracted to determine the concentration of purified RL produced at each time point.

**Fig. 1:**
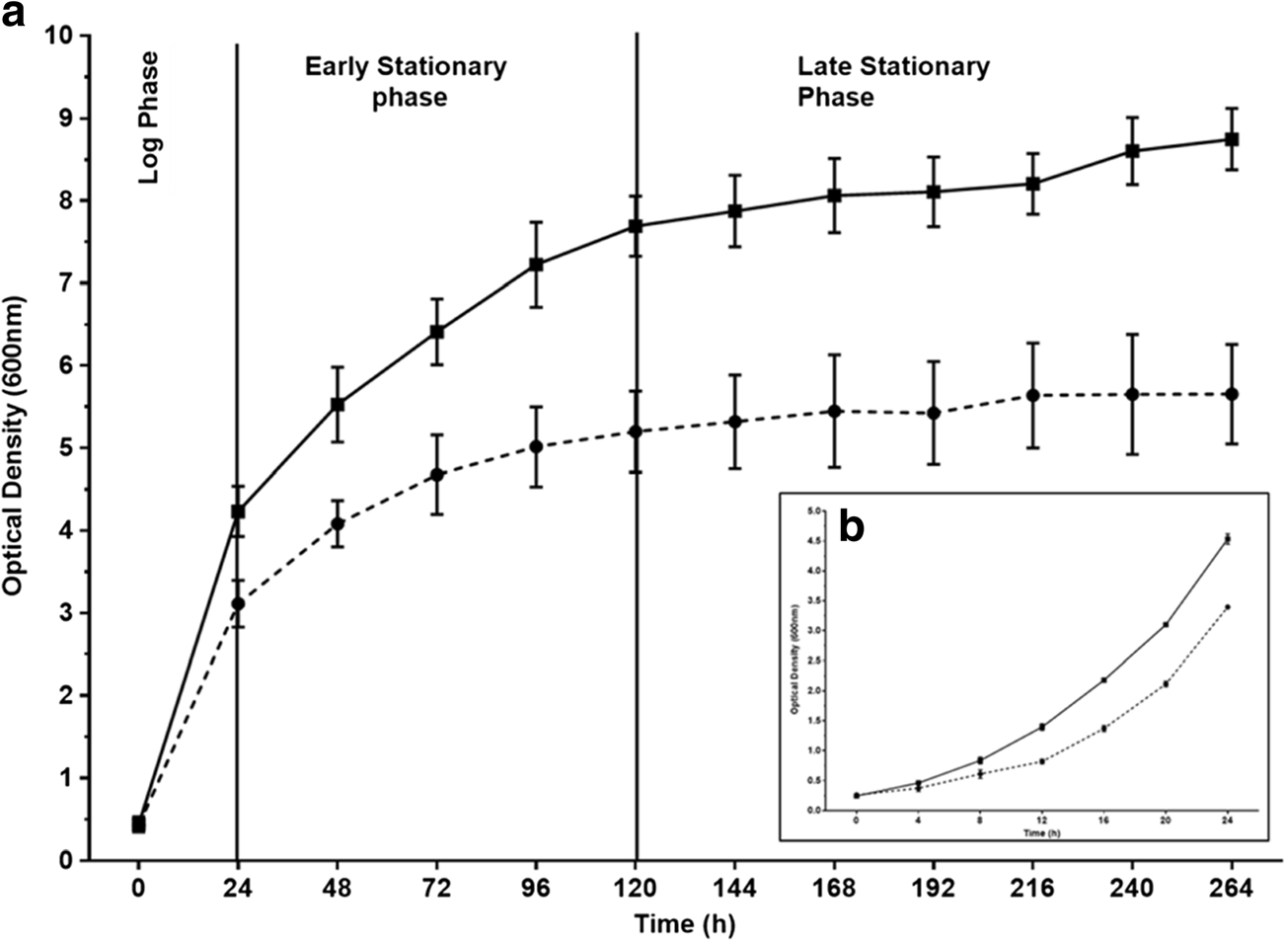
**a** Growth of *B. thailandensis* E264 in NB +4 % glycerol, 200 rpm rotary shaking at two different incubation temperatures. One culture was incubated at 25 °C (*dashed line*) and the other incubated at 30 °C (*solid line*). Data is presented as optical density (OD) values measured at 600 nm. Samples were taken at 24-h intervals throughout the growth period. **b** Log phase of growth within the first 24 h of fermentations

The *B. thailandensis* E264 culture grown at 30 °C generated a larger dry cell biomass throughout the growth period with 7.71 g/l after 264 h compared to 4.75 g/l for the 25 °C culture after the same time. Both cultures followed the same growth pattern with the exponential/log phase occurring within the first 24 h, and an early stationary phase was then observed between 24 and 120 h where growth of the bacterium in each culture continued at a slower rate. From 120 h onwards, both cultures entered a late stationary phase where cell growth had almost completely stopped and OD_600_ values either remained stable or increased very slowly (Fig. 1).

**Fig. 2:**
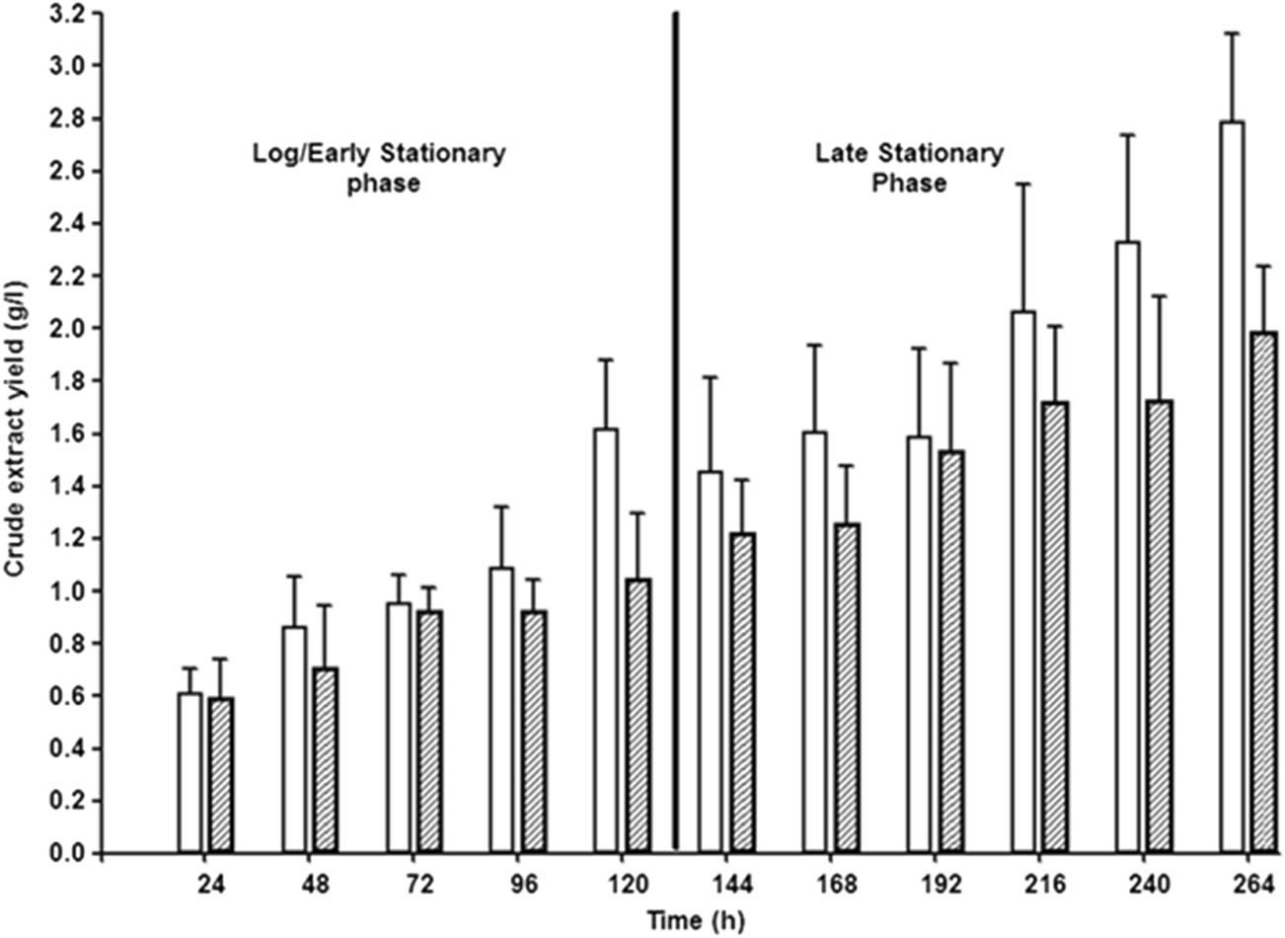
Crude extract concentrations from cell-free supernatants throughout the growth period. This shows the difference in rhamnolipid production by *B. thailandensis* E264 when incubated at 25 °C (*clear bars*) compared to 30 °C (*dashed bars*). For each time point, acid precipitation and solvent extraction were carried out on a 50-ml sample of cell-free supernatant. The resulting product was weighed and used to calculate the production concentration in grammes per litre

The *B. thailandensis* E264 culture grown at 25 °C consistently produced a higher concentration of crude RL extract than the culture grown at 30 °C. After 264-h incubation, the mean concentration produced by the culture grown at 25 °C was 2.79 g/l, whereas the respective mean for the 30 °C culture was 1.99 g/l (Fig. 2). This difference in crude RL extract concentration was observed throughout the incubation period; however, it was most noticeable in the final 72 h. The rate of RL production after 264 h was calculated as 0.258 g/g dry cell biomass for the 30 °C culture compared to 0.587 g/g dry cell biomass for the 25 °C culture. Gravimetric data obtained before and after SPE showed that the initial crude extracts were composed of between 15 and 25 % contaminants (data not shown). This was consistent throughout the fermentation studies and across the various experimental conditions carried out.

### Separation and analysis of the crude extract produced by *B. thailandensis* E264

The crude extracts produced by *B. thailandensis* E264 were initially analysed by direct infusion ESI-MS. The mass spectrum of the crude extract showed a dominant peak representing a pseudomolecular ion of 761 m/z; this corresponds to the Di-RL C14-C14. This was also observed in previous work (Dubeau et al. 2009). In addition, there were significant peaks at 705, 733 and 789 m/z which correspond to the Di-RL congeners Di-RL C12-C12, Di-RL C12-C14/C14-C12 and Di-RL C14-C16/C16-C14. The Mono-RL C14-C14 was also observed in the mass spectrum at a peak of 615 m/z; however, this was at a low relative abundance.

**Fig. 3:**
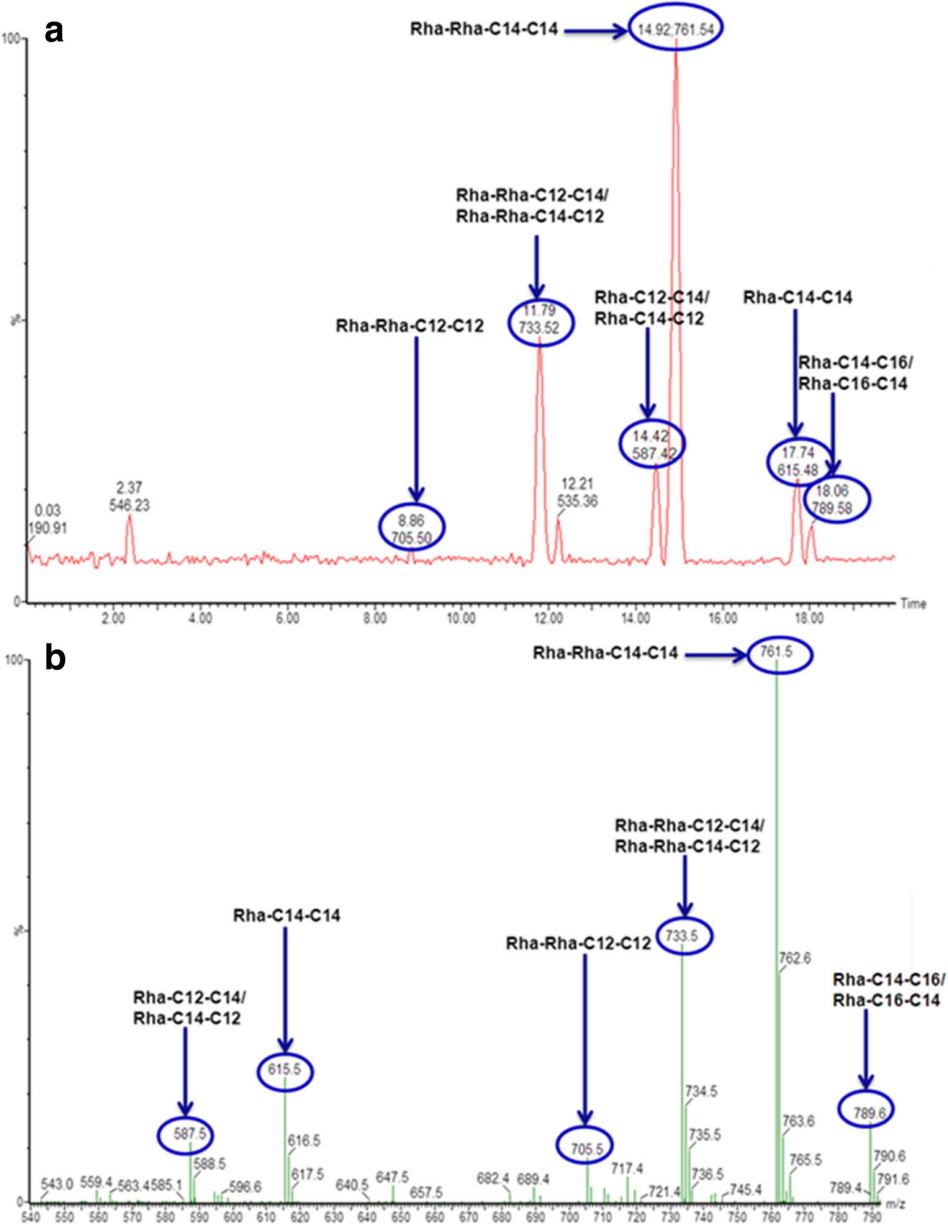
Separation of rhamnolipid congeners produced by *B. thailandensis* E264. **a** LC-QToF-MS of rhamnolipid crude extract after SPE purification: Static phase, Aglient poroshell SB-C3, 2.1 × 100 mm, particle size 2.7 μm. Mobile phase 1, H_2_O (4 mM ammonium acetate), and mobile phase 2, MeCN, were used for chromatographic separation as follows: 0–17 min 50–70 % mobile phase 2, 17.0–17.5 min 70 % mobile phase 2, 17.5–18.0 min: 70–50 % mobile phase 2, 18–20 min: 50 % mobile phase 2. **b** QToF-MS profile of rhamnolipid crude extract after SPE purification. SPE purification was carried out using Strata SI-1 silica (55 μm, 70 A) 2 g/12 ml Giga Tubes. CHCl_3_ was used to clean the column before the sample was added. The sample was then dissolved in CHCl_3_ and added to the column, and pure CHCl_3_ was ran through the column to clean and remove any unwanted products from the sample. Finally, the purified rhamnolipid was eluted using a 1:1 *v*/*v* solution of CHCl_3_/MeOH

In order to give a more quantitative image of the RLs produced by *B. thailandensis* E264, LC-QToF-MS was used to separate the individual congeners present in the crude extract (Fig. 3). Solid phase extraction (SPE) was also used to remove any impurities from the sample prior to analysis. The trace obtained from HPLC-MS showed a good separation of congeners, and, as expected, Di-RL C14-C14 was observed in the highest abundance (Table 2).

**Table 2:** Relative abundance of specific rhamnolipids produced by *B. thailandensis* E264

Rhamnolipid congener	Pseudomolecular ion (m/z)	Retention time	Relative abundance (%)
Rha-C12-C12	559	11.25	0.82
Rha-C12-C14/C14-C12	587	14.42	5.76
Rha-C14-C14	615	17.74	11.51
Rha-Rha-C12-C12	705	8.96	2.56
Rha-Rha-C12-C14/C14-C12	733	11.79	16.87
Rha-Rha-C14-C14	761	14.92	41.88
Rha-Rha-C14-C16/C16-C14	789	18.06	8.14

### Expression of the *rhlA*, *rhlB* and *rhlC* genes throughout the *B. thailandensis* E264 growth curve

**Fig. 4:**
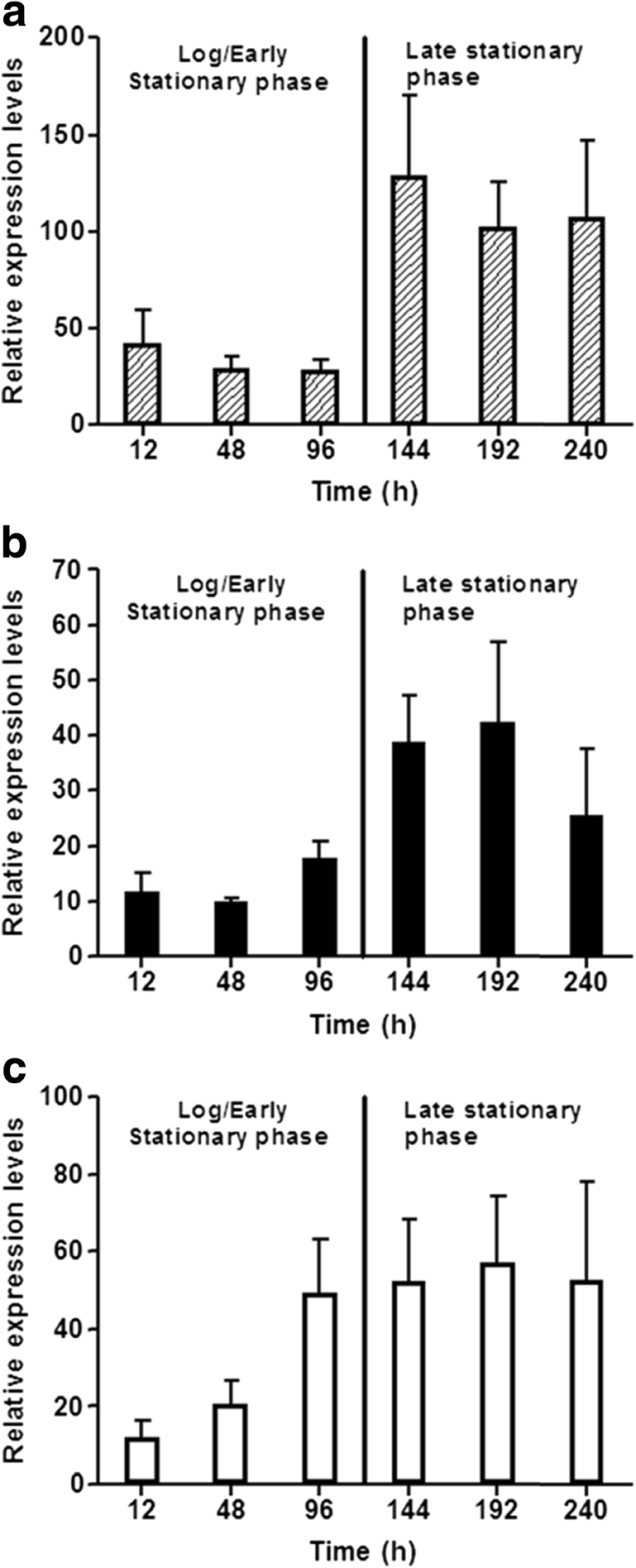
qPCR gene expression profiles of the *rhlA* (*dashed bars*) (**a**), *rhlB* (*black bars*) (**b**) and *rhlC* (*clear bars*) (**c**) genes in *B. thailandensis* E264. Gene expression data was generated by growing *B. thailandensis* E264 at 25 °C, 200 rpm rotary shaking in NB + 4 % glycerol for a total of 264 h. Cell pellets were taken at various time points throughout the growth period and total RNA was extracted from them. cDNA was then made and used for qPCR analysis

In order to generate a better understanding of RL biosynthesis in *B. thailandensis* E264, a gene expression profile was generated using qPCR throughout the incubation period at 25 °C, as this showed the highest levels of crude extract production. Internal primers specific to the *rhlA*, *rhlB* and *rhlC* genes were designed and used to give an overall expression profile of the RL biosynthesis genes. The geometric mean expression levels of three commonly used reference genes *rpoB*, *rpoD* and *gyrB* were used to quantify gene expression. All qPCR methods and protocols were carried out according to the MIQE guidelines (Bustin et al. 2009). Results showed that in the exponential growth phase, all three RL production genes were regulated at low levels; however, after 96 h, the *rhlC* gene was upregulated whilst the expression levels of *rhlA* and *rhlB* remained low (Fig. 4). At the onset of late stationary phase (>144 h), the expression levels of all three RL biosynthesis genes had increased and all remained elevated until the end of the fermentation (264 h). Expression of all three RL biosynthesis genes continued throughout the fermentation. When expression levels of each gene were compared, it was observed that the *rhlA* gene is expressed at a much higher level than *rhlB* and *rhlC*.

### Analysis of RL production by *B. thailandensis* E264 in a 5-l bioreactor under controlled conditions

A 5-l bioreactor system was used (working volume of 4 l) in order to determine whether RL production in *B. thailandensis* E264 is maintained when the fermentation is scaled up. Bacterial growth was monitored throughout the fermentation by measuring the OD_600_ at 24-h intervals for a total of 264 h. The incubation temperature was set to 25 °C as this was previously shown to be a better temperature for RL production than 30 °C. Aeration conditions were programmed to maintain a DO_2_ level of 20 % which was maintained by the rate of stirring (set between 50 and 500 rpm). Fermentations were carried out in biological duplicate.

**Fig. 5:**
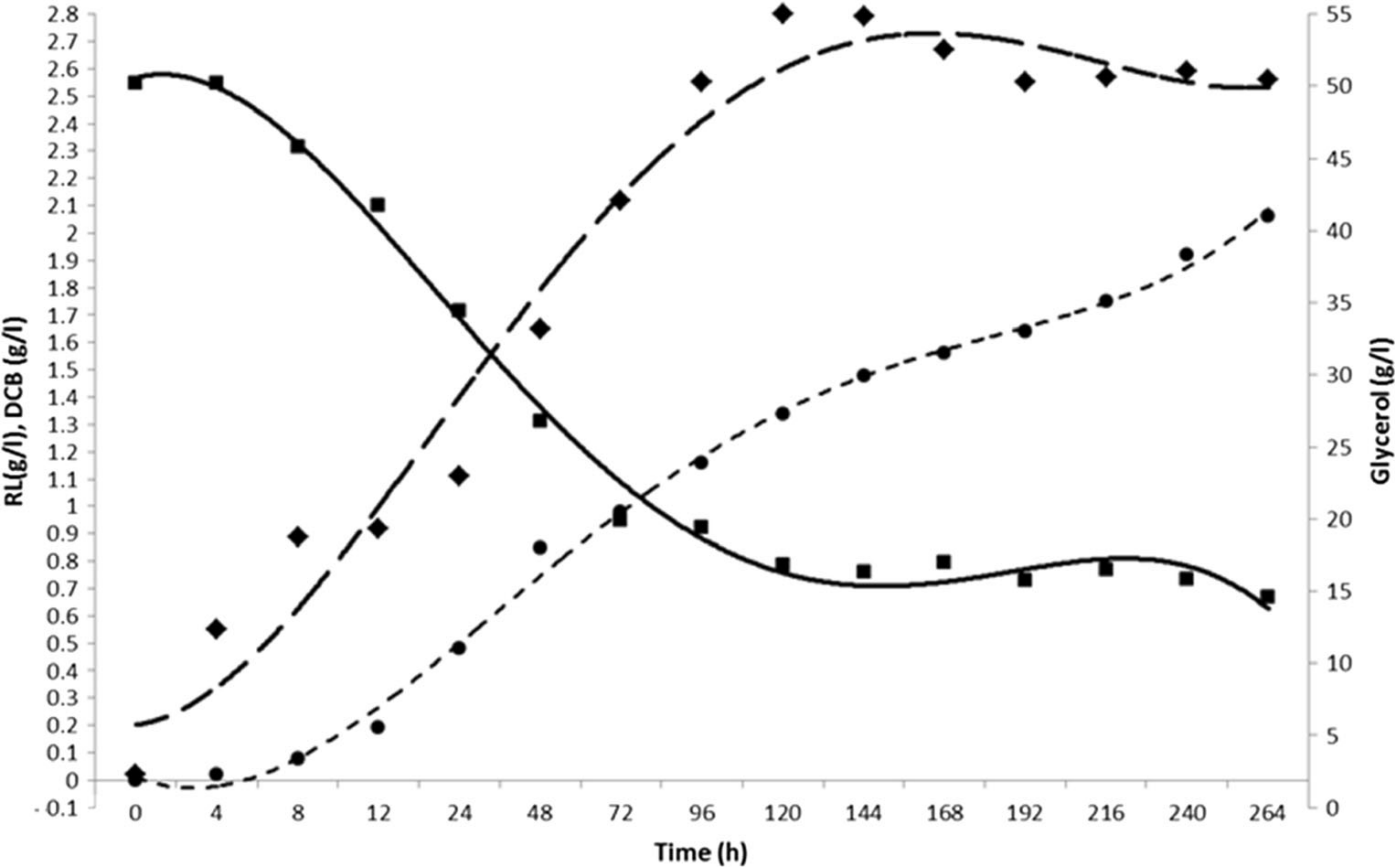
The growth of *B. thailandensis* E264 (DCB) (*black diamond*) in a 5-l bioreactor with a 4-l working volume incubated at 25 °C with 20 % dO_2_ controlled by a stirring rate of 50–500 rpm. Plotted against RL production (g/l) (*black circle*), glycerol depletion (g/l) (*black square*) throughout the growth period in NB + 4 % glycerol

**Fig. 6:**
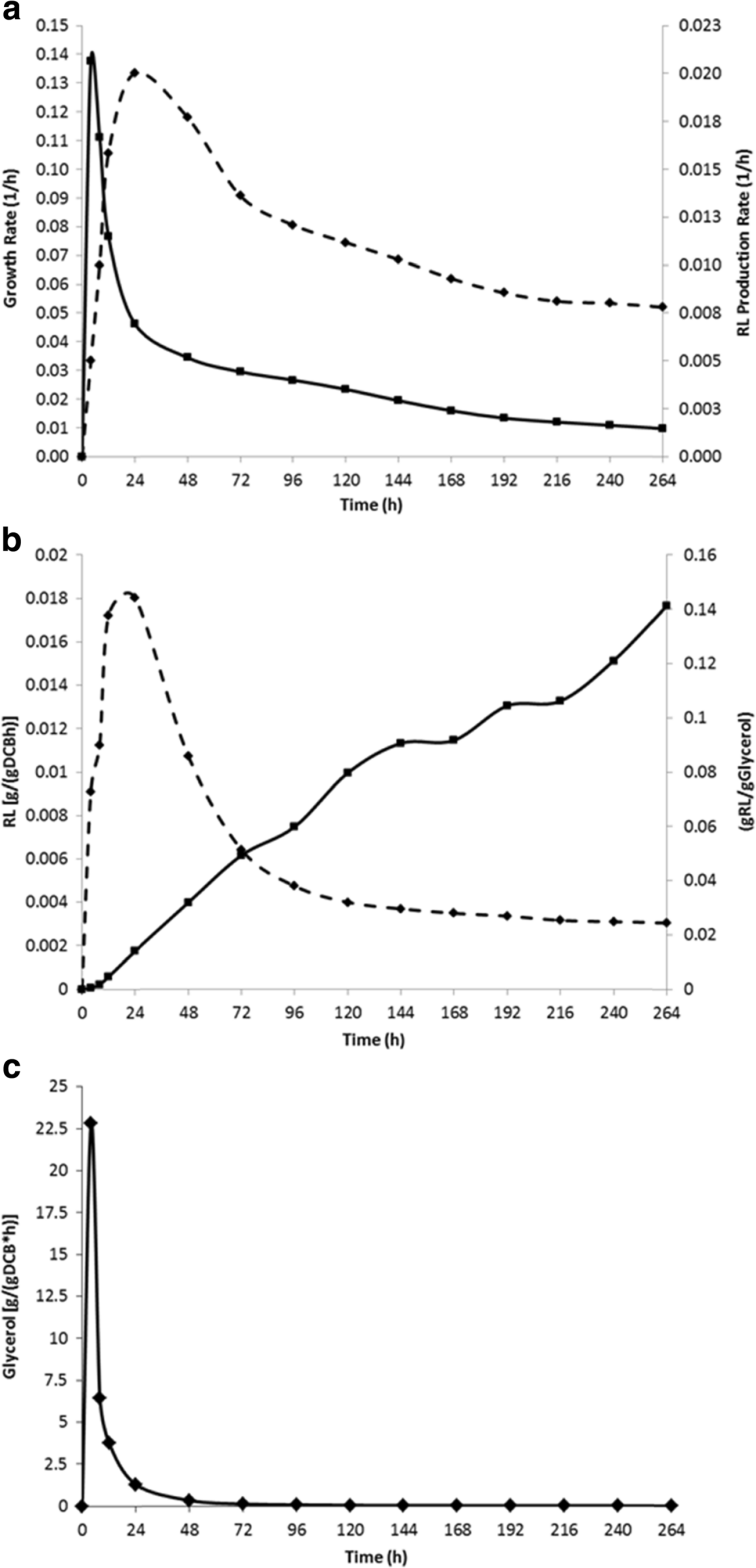
Analysis of *B. thailandensis* E264 grown in a 5-l bioreactor with a 4-l working volume incubated at 25 °C with 20 % dO_2_ controlled by a stirring rate of 50–500 rpm in NB + 4 % glycerol. **a** Comparison of growth rate (1/h) (*black square*) and RL production rate (1/h) (*black circle*) during the fermentation period. **b** Biomass-specific RL production rate [gRL/(gDCB * h)] and substrate to product conversion yields (gRL/gGlycerol) obtained throughout the fermentation period. **c** Glycerol uptake rate [g/(gDCB * h)] during the fermentation period

Results from the OD_600_ measurements in the bioreactor showed that *B. thailandensis* E264 followed a similar growth pattern to that seen in the shake flask experiments (Fig. 5). Glycerol depletion was also measured throughout the incubation period to quantify how much glycerol was consumed and also to determine the stage at which glycerol consumption is at its highest. Using the colorimetric assay for glycerol (see “Methods and materials”), results showed an initial concentration of 50.1 g/l at 0 h which was reduced to 16.8 g/l after 120 h, and this exponential decrease correlated with the log and early stationary phases of growth where almost all cell biomass was accumulated. Between 120 and 264 h (late stationary phase), the glycerol concentration continued to decrease but at a much slower rate, from 16.84 to 14.58 g/l. Analysis of the substrate (glycerol) uptake rate (Fig 6) showed that a significant spike during the initial 24-h period (peaking at 22.817 g/(gDCB * h)) after which there was a gradual decrease throughout the rest of the fermentation period with a final value of 0.022 after 264-h incubation. This pattern of substrate uptake correlated with the bacterial growth rate in which there was also an initial spike during the first 24 h (peaking at 0.1375 1/h) of the fermentation (Fig 6). At around 120 h into the fermentation, a significant amount of foam was produced from each culture, and head space was left in the vessel to accommodate for this as well as an external aseptic foam trap. The mean concentration of crude RL extract obtained after 264 h was 2.06 g/l; however, as some of the cell biomass was lost through foaming, this value may have been higher had it remained in the incubation vessel. Further analysis of the fermentation showed that the rate of RL production was highest during the first 72 h (0.020 1/h) and steadily decreased throughout the remainder of the run. Substrate to product conversion yield steadily increased throughout the growth period in a linear fashion with the highest value of 0.141 gRL/gGlycerol observed after 264 h. The DCB-specific rate of RL production was also calculated over the course of the fermentation and showed a peak of 0.018 gRL/(gDCB * h) during the log/early stationary phase in the first 48 h; this then decreased as the levels of DCB increased during the late stationary phase with a final value of 0.003 gRL/(gDCB * h).

### BLASTp alignments of *rhlA*, *rhlB* and *rhlC* amino acid sequences against the *P. aeruginosa* genome

The BLASTp algorithm was used to determine the level of homology between amino acid sequence coding for the *rhlA*, *rhlB* and *rhlC* genes in *B. thailandensis* and their corresponding orthologs in *P. aeruginosa*. Results showed that there were sequence identities of less than 50 % in all three *rhl* genes between these two species. As the *B. thailandensis* E264 genome contains two copies of each gene, all six genes were used in the alignment analysis.

## Discussion

After studying the RL production in *B. thailandensis* E264, it is clear that there are some interesting genotypic and phenotypic differences between this bacterium and the well-studied RL producer *P. aeruginosa*. When RL production was first described in *B. thailandensis* E264, it was also highlighted that it has two identical gene clusters containing copies of the RL synthesis genes (*rhlA*, *rhlB* and *rhlC*) present at different sites in its genome (Dubeau et al. 2009). This is unlike *P. aeruginosa* which only has one copy of each gene with the *rhlA* and *rhlB* genes located in the same operon whilst *rhlC* is located separately (Ochsner et al. 1994; Rahim et al. 2001). In addition, this study has shown that there are significant differences in the amino acid sequences of the *rhlA*, *rhlB* and *rhlC* genes between these two bacterial species (Table 3). This could potentially account for the structural differences seen between specific RL congeners produced by *B. thailandensis* E264 and *P. aeruginosa*. It has been previously reported that the main congeners produced by *P. aeruginosa* are a combination of mono- and di-RLs at ratios ranging from 1:1 to 3.4:1 (di-RL/mono-RL) with the Di-RL C10-C10 consistently produced in most abundance (Abdel-Mawgoud et al. 2010; Müller et al. 2010). In addition, there has been a wide range of RL congeners previously reported to be produced by *P. aeruginosa* at lower levels (Lotfabad et al. 2010; Mueller et al. 2011, Abdel-Mawgoud et al. 2010). However, in contrast to this, the variation of RL congeners produced by *B. thailandensis* E264 seems to be low with only six congeners produced in significant quantities throughout this study (Fig. 3). The Di-RL C14-C14 was observed in the highest abundance (41.88 %) followed by Di-RL C12-C14/C14-C12 (16.7 %) and Di-RL C14-C16/C16-C14 (8.14 %). Using the relative abundance values, the ratio of di-RL to mono-RL was calculated at 3.84:1 showing that *B. thailandensis* E264 is more efficient in di-RL production that of *P. aeruginosa*. The crude extract obtained using the methods described here shows that a relatively pure product was obtained with low congener variation; this means that on an industrial scale, there would be less downstream processing required to generate a pure RL product from cell-free supernatant; however, more optimisation is still required in order to increase the rate of production to an industrially relevant level. Whilst it would be ideal to show a direct quantitative comparison between RLs produced by *P. aeruginosa* in previous studies and those produced by *B. thailandensis* in this study, the comparison would not be truly representative of the variation. This is due to the fact that many of the methods used in the literature for the detection and quantification of RLs are both indirect and/or inaccurate leading to significant inaccuracies in the claims of RL concentrations produced (an issue previously discussed by Marchant et al. (2014)). This study has used an LC-MS method developed specifically for RLs produced by *B. thailandensis* to ensure high levels of accuracy were achieved.

**Table 3:** Table showing amino acid sequence BLASTp alignments from the *rhlA*, *rhlB* and *rhlC* genes found in *B. thailandensis* E264 against the *P. aeruginosa* genome

*B. thailandensis* E264 gene information		BLASTp against *Pseudomonas aeruginosa* taxid: 287			
Gene name	Locus tag	Protein name/function	Query coverage (%)	% identity	Accession no.
*rhlA*	BTH_II1075	Rhamnosyltransferase 1 subunit a [*Pseudomonas aeruginosa*]	89	48	WP_016263193.1
*rhlB*	BTH_II1076	Glycosyl transferase family 1 [*Pseudomonas aeruginosa*]	85	49	WP_024914755.1
*rhlC*	BTH_II1079	Glycosyl transferase family 2 [*Pseudomonas aeruginosa*]	95	46	WP_009876069.1
*rhlC*	BTH_II1877	Glycosyl transferase family 2 [*Pseudomonas aeruginosa*]	95	46	WP_009876069.1
*rhlB*	BTH_II1880	Glycosyl transferase family 1 [*Pseudomonas aeruginosa*]	85	49	WP_024914755.1
*rhlA*	BTH_II1881	Rhamnosyltransferase 1 subunit a [*Pseudomonas aeruginosa*]	89	48	WP_016263193.1

From the growth curve analysis that was carried out, it is clear that *B. thailandensis* E264 is quite a slow growing bacterium that can remain in the stationary phase of growth for extended periods of time. This is not surprising however as many soil bacteria have adapted to survive through extended periods of cell stress and nutrient starvation (Jørgensen et al. 1999, Swiecilo and Zych-Wezyk 2013). In the closely related *B. pseudomallei*, it was shown that the expression of the general cell stress response regulator, *rpoS*, is upregulated upon entry into the stationary phase of growth (Subsin et al. 2003). This is responsible for inducing the expression of various virulence factors in many gram-negative bacteria as well as causing significant shifts in global gene expression levels (Suh et al. 1999; Kang et al. 2008; Dong and Schellhorn 2010).

One of the significant findings of this study was that *B. thailandensis* E264 consistently produced a higher concentration of RL containing crude extract throughout the growth period when incubated at 25 °C compared to 30 °C. What is interesting about this is that when incubated at 30 °C, a significantly higher level of biomass was generated; however, this correlated with the lower level of RL production. Direct infusion ESI-MS was carried out on all crude extract samples obtained (data not shown), and each showed an almost identical mass spectrum indicating that the production of specific RL congeners is constant throughout the growth period. There are a number of factors that could explain the higher rate of RL production at a lower incubation temperature. For example, when growing at a lower metabolic rate, there may have been more free carbon within the cell for production of metabolites such as RLs. Another explanation may be that the optimal temperature for enzyme activity of the rhamnosyltransferases in *B. thailandensis* E264 may be lower than the optimal growth temperature. This is quite common in bacterial strains that have been isolated from soils and water sources in the environment (Choo et al. 1998; Morita et al. 1997). What is important to note is that the concentration of RLs produced using the small scale shake flask was maintained when the fermentation process was scaled up to a working volume of 4 l and carried out in a bioreactor under controlled conditions. Figure 5 shows that the same growth pattern was also maintained in the large scale fermentation whilst the concentration of DCB was lower than that seen in the shake flask experiments. Analysis of glycerol depletion was also carried out during the scaled up fermentation and showed an almost inversion of the cell growth pattern. Substrate uptake rate (Fig. 6) showed that a significant amount of the substrate was used for cell growth during the log and early stationary phases, and when *B. thailandensis* entered the stationary phase, the rate of substrate consumption decreased along with the growth rate of the bacteria. Another important point was that the concentration of glycerol in the fermentation media was never depleted and instead RL production rate slowed. This may indicate the presence of a metabolic control system that will slow metabolic activity when carbon substrate concentrations drop below a threshold level such as that observed in *E. coli* (Nikel et al. 2009).

In order to determine expression levels of the RL synthesis genes (*rhlA*, *rhlB* and *rhlC*) in *B. thailandensis* E264, qPCR was carried out using a combination of the commonly used reference genes *rpoB*, *rpoD* and *gyrB* whose expression levels are known to remain at a constant level and are not affected by different experimental factors (Kozera and Rapacz 2013). Expression levels of all three RL synthesis genes remained low in the log/early stationary phase. After 96 h, the *rhlC* gene was upregulated, and after 144 h, expression levels of the *rhlA* and *rhlB* genes had also increased (Fig. 4). Once in late stationary phase, the expression of all three RL synthesis genes remained elevated for the remainder of the growth period. This correlated with the level of RL production as it continued to increase throughout the growth period; however, further analysis showed an increased rate of RL production during the log/early stationary phase compared to the late stationary phase. This was interesting as *rhl*A and *rhl*B expression levels were lower during this period and may suggest that post-transcriptional control systems (i.e. quorum sensing) may be affecting RL production rates during the fermentation period. As *B. thailandensis* E264 contains two identical gene clusters containing copies of the RL synthesis genes, the levels of expression reported here do not show the expression of single genes but rather the overall expression of two copies of the same gene. It must be noted that it is still unclear as to whether these two gene clusters are controlled by the same promoter/regulator system. By looking at the gene expression data, it is evident that expression of the RL synthesis genes is continuous at a low level and is increased upon entry to/in preparation for late stationary phase. One hypothesis for this expression pattern could be that one of the RL gene clusters is continuously expressed at a low level whereas expression of other is induced (from no expression to a high level of expression) by either quorum sensing (as seen in *P. aeruginosa*), the stationary phase sigma factor RpoS or a combination of both (Wongtrakoongate et al. 2012, Subsin et al. 2003, Solis et al. 2006). A recent study by Perfumo et al. (2013)) showed that the RL gene expression pattern in *P. aeruginosa* is significantly different than that of *B. thailandensis* in that the expression of the rhlA and rhlB genes is upregulated during the log and early stationary phase with the rhlC gene showing only low levels of expression during this period. At the onset of stationary phase, there is a shift in the expression pattern where the rhlA and rhlB genes are downregulated and the rhlC gene is significantly upregulated (Perfumo et al. 2013). This shows that RL production in *P. aeruginosa* is limited by the complex cell density dependant systems that have been previously characterised in *P. aeruginosa* (Pearson et al. 1997; Dekimpe and Deziel 2009; Reis et al. 2011). This study has shown that control of the RL biosynthesis genes in *B. thailandensis* E264 does not follow this pattern and that the continued gene expression of *rhl*A, *rhl*B and *rhl*C could potentially allow for longer and more sustained periods of RL production in *B. thailandensis*. This could potentially lead to the development of effective immobilised cell fermentations and/or continuous fermentation systems that would not be viable when using *P. aeruginosa* as the RL-producing organism.

It is clear that RL production in *B. thailandensis* E264 shows some interesting patterns and characteristics and therefore makes it an excellent candidate for further study as a potential alternative to *P. aeruginosa* for RL production on an industrial scale.
